# Patient handling system for carbon ion beam scanning therapy

**DOI:** 10.1120/jacmp.v13i6.3926

**Published:** 2012-11-08

**Authors:** Shinichiro Mori, Toshiyuki Shirai, Yuka Takei, Takuji Furukawa, Taku Inaniwa, Yuka Matsuzaki, Motoki Kumagai, Takeshi Murakami, Koji Noda

**Affiliations:** ^1^ Research Center for Charged Particle Therapy National Institute of Radiological Sciences Inage‐ku Japan

**Keywords:** carbon ion beam scanning, particle therapy, treatment workflow, patient handling, imaging

## Abstract

Our institution established a new treatment facility for carbon ion beam scanning therapy in 2010. The major advantages of scanning beam treatment compared to the passive beam treatment are the following: high dose conformation with less excessive dose to the normal tissues, no bolus compensator and patient collimator/ multi‐leaf collimator, better dose efficiency by reducing the number of scatters. The new facility was designed to solve several problems encountered in the existing facility, at which several thousand patients were treated over more than 15 years. Here, we introduce the patient handling system in the new treatment facility. The new facility incorporates three main systems, a scanning irradiation system (S‐IR), treatment planning system (TPS), and patient handling system (PTH). The PTH covers a wide range of functions including imaging, geometrical/position accuracy including motion management (immobilization, robotic arm treatment bed), layout of the treatment room, treatment workflow, software, and others. The first clinical trials without respiratory gating have been successfully started. The PTH allows a reduction in patient stay in the treatment room to as few as 7 min. The PTH plays an important role in carbon ion beam scanning therapy at the new institution, particularly in the management of patient handling, application of image‐guided therapy, and improvement of treatment workflow, and thereby allows substantially better treatment at minimum cost.

PACS numbers: 87.56.‐v; 87.57.‐s; 87.55.‐x

## I. INTRODUCTION

Rapid technological advances in hardware/software over the past few years have led to new applications in both the photon and particle beam fields. To take one example, the availability of imaging modalities has expanded from three‐dimensional (3D) to four‐dimensional (4D) using CT, MRI, and PET.,. Further, the evolution of treatment planning has also extended to 4D with deformable image registration.^1^, ^2^ The superior dose conformation of particles over photon beams has now seen the establishment of more than 28 particle treatment centers worldwide, including three carbon ion beam centers, and the ongoing construction of more. Our center recently constructed a new treatment facility for carbon ion beam scanning treatment as an extension of the existing treatment building, which provides passive beam irradiation. The new treatment facility is designed to facilitate the integration of several systems with the overall goal of increasing treatment accuracy and improving treatment workflow in terms of their physical, technical, and clinical aspects. While the irradiation and treatment planning systems have been reported elsewhere,^3^, ^4^ the patient handling system (PTH) has not been described. Here, we introduc the PTH in the new treatment facility at our center and describe its current status.

## II. MATERIALS AND METHODS

### A. New treatment facility

**Figure 1:**
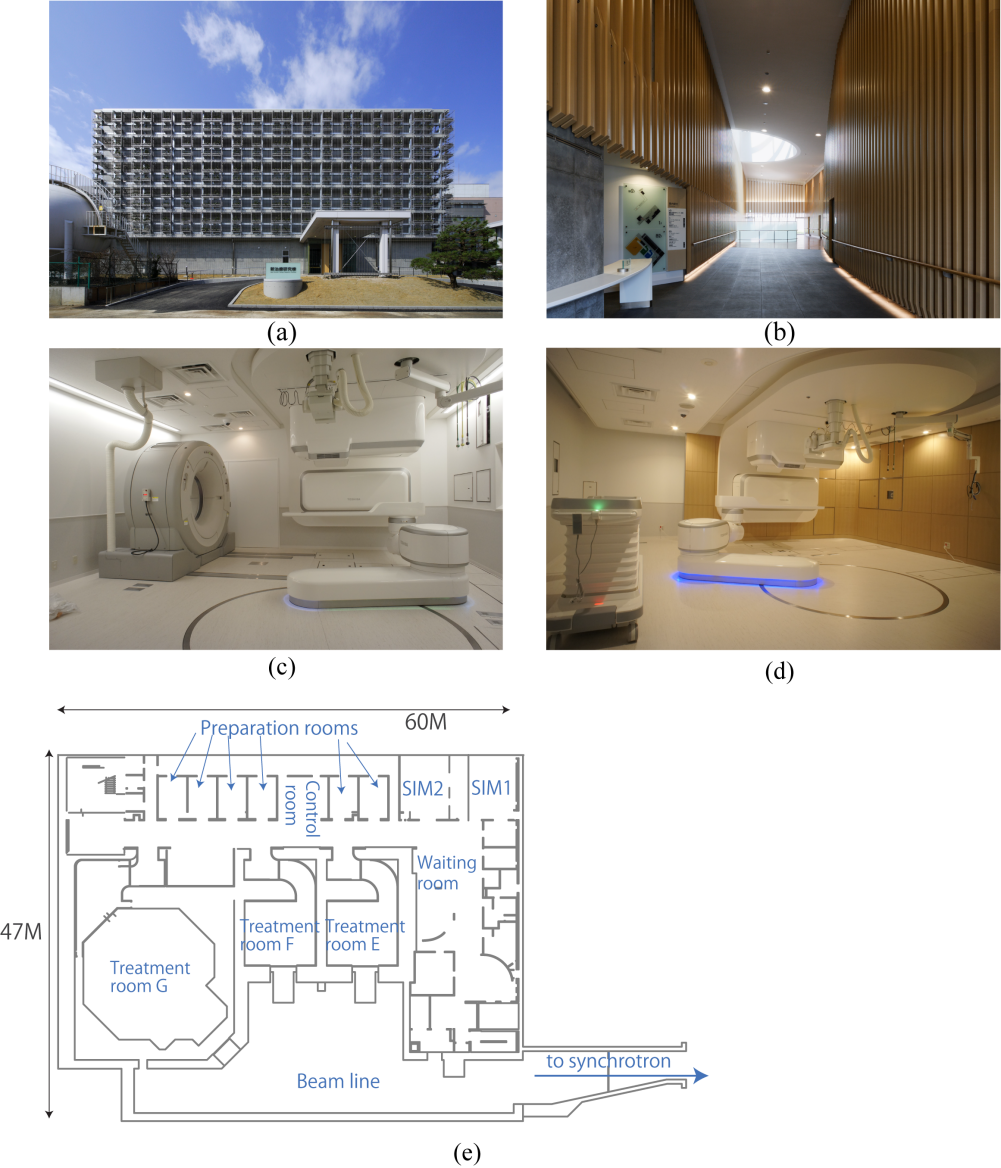
Front view (a) of the new treatment facility building; (b) entrance view; (c) simulation room; (d) treatment room; (e) 2nd basement map.

The new treatment facility was constructed as an extension of the existing treatment building and shared the accelerator with it.^5^, ^6^ The goal of the new facility was to provide carbon ion beam scanning irradiation with respiratory gating. It was designed to treat more than 1000 patients per year, and to solve a number of problems encountered in passive beam therapy, including expensive patient accessories (compensating bolus, patient collimator, disposal immobilization devices), limitations in patient poses for CT acquisition (standard bore size CT scanner), short treatment table, collision accidents between the imaging device and patient, and limitations in patient treatment numbers, as detailed in the next section. Construction was started in February 2009 and completed in March 2010 ((Figs. 1a)and (1b). The new facility occupies the 2nd basement level of the original facility and consists of two simulation rooms ((Fig. 1c), three treatment rooms (two fixed beam ports ((Fig. 1d) and one gantry port^(7)^), and six preparation rooms ((Fig. 1e). Installation of treatment equipment was started in June 2010 and commissioning was completed in March 2011. The Tohoku earthquake (magnitude 9.0 Mw), one of the five most powerful earthquakes recorded since 1900, hit Japan on 11th March 2011,^(8)^ delaying the start of treatment until May 2011.

**Figure 2:**
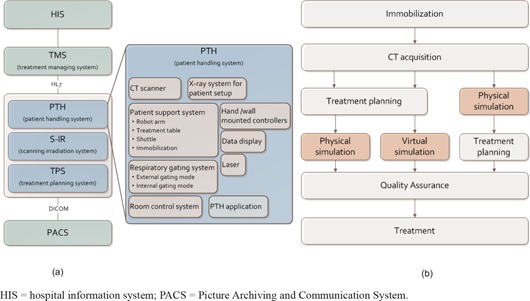
Overview (a) of the new treatment facility system; (b) three main types of treatment workflow.

The new facility is organized into three main systems, a Scanning Irradiation System (S‐IR), a Treatment Planning System (TPS), and a Patient Handling System (PTH) ((Fig. 2a). The S‐IR manages the irradiation hardware/software (including scanning beam control and delivery systems) and its corresponding data information. This system allows tracking any downtime of beam delivery and hardware failures. The scanning irradiation technique does not require a compensating bolus, patient collimator, or multileaf collimator. As a result, a few times for changing these accessories including therapists walking in/out treatment room to bring them could be minimized in treatment workflow. Moreover, it avoids falling accident of accessories especially in vertical irradiation port. Irradiation depth (= beam range) is changed by the range shifter and synchrotron energy change.^9^, ^10^ Delivery of a physical dose of 1 Gy to a 60 mm diameter ball with eight rescans takes 18 s (including range shifter change time of 8 s) for range shifter scanning strategy.^(3)^ We did not describe the comparison of the irradiation time in between scanning and passive beam irradiations because it is strongly depended on the target shape and irradiation conditions. The horizontal port in the simulation room has been moved back from the original position to allow sufficient space for therapist operations. The TPS includes a carbon ion beam scanning dose calculation engine with a biological effect, and intensity‐modulated carbon ion therapy is available.^(4)^

In contrast, the PTH covers a wide range of functions, including imaging, geometrical/position accuracy including motion management, layout of the treatment room, and treatment workflow. The treatment management system (TMS) transfers patient information and order messages from the hospital information system (HIS), and exchanges order messages/responses among the three systems in health level 7 (HL7) format ((Fig. 2a). These systems have several units which interactively exchange data.

HIS=hospital information system; PACS=Picture Archiving and Communication System.

### B. Patient handling system

The main purpose of the PTH is the acquisition of patient images, maintenance of geometrical accuracy, motion management, and improvement of treatment workflow throughout the treatment course. The PTH includes several modalities and software to support treatment (Fig. 2).

#### B.1 Patient support system

**Figure 3:**
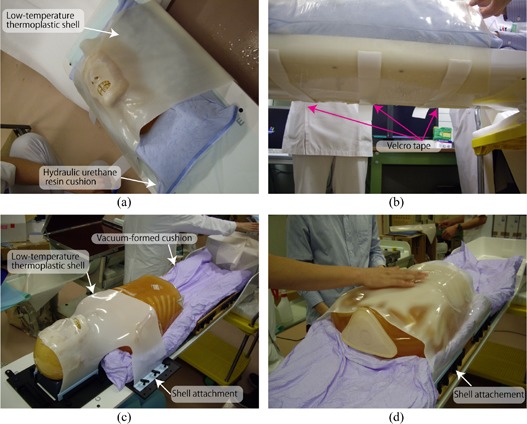
Example of immobilization for head treatment (a) using a hydraulic urethane resin cushion and low‐temperature thermoplastic shell; (b) a shell device is taped at the bottom of the table; (c) example of immobilization for head treatment using a vacuum‐formed cushion and shell; (d) example of immobilization for thoracic/upper abdominal treatment using a vacuum‐formed cushion and shell.

*Immobilization*: Since the treatment table can be rotated to allow a greater range of beam angles (approximately ±20° roll at a maximum), immobilization in passive irradiation treatment (= orthogonal fixed beam ports) is done with a relative thicker shell (3 mm thickness) made of a low‐temperature thermoplastic (Shellfitter; Keraray Co., Ltd., Osaka, Japan) and hydraulic urethane resin (Moldcare, Alcare, Tokyo, Japan). The shell device is fixed by taping to the table bottom, with tightening or loosening adjustment as required ((Figs. 3a)and (3b). More recently, however, we have started to use several types of reusable immobilization to reduce costs. For example, a vacuum‐formed cushion (ESFORM, Engineering System, Nagano, Japan) is used to fix partial or whole‐body positioning, and shell device is attached to the side of the base plate ((Figs. 3c)and (3d). The new facility has also introduced several other devices, such as an arm support and a foot rest.

**Figure 4:**
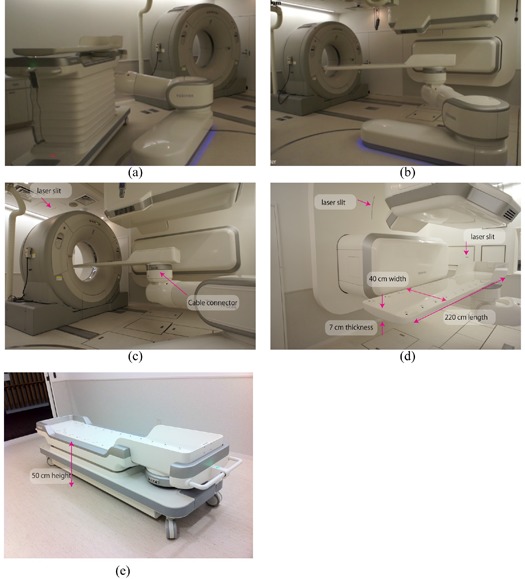
Connection of the robotic arm (a) from the shuttle with the treatment table; (b) transfer of the treatment table to the isocenter by the robotic arm; (c) CT acquisition position; (d) treatment table; (e) shuttle‐down position.

*Robot arm treatment bed*: A robot arm positioning system is installed at several particle beam centers.^11^, ^13^ The robot arm transfers the patient to the treatment table with six degrees of freedom (the table top coordinate system is described in IEC^(14)^). As part of this equipment, we developed a SCARA (selective compliance assembly robot arm)‐type robotic arm in collaboration with Toshiba Corporation ((Figs. 1c), 1(d) and 4(a)–4(d)).

Linear movements are 2400 mm in the longitudinal direction ((Fig. 4b), 600 mm in the vertical, which can be extended depending on the application, and ± 300 mm in the lateral direction. Rotational movements are ‐15° to 195° of rotation (isocentric rotation), ±20° of roll, and ±5° of pitch. Maximum tolerated load is 150 kg. Regarding accuracy, absolute and relative position accuracies are within the range of a sphere of 0.5 mm and 0.3 mm diameter, respectively, as measured by a laser tracker system without various loads. The robot arm treatment bed was integrated with auto deflection correction, although we do not currently use this function. To ensure patient comfort, the robot arm transfers the patient on a smoothed orbit with acceleration and deceleration, and rotates at the center of the patient head region. To improve the safety of controls, the robot arm is equipped with noncontact sensors integrated at the side of the robot arms to avoid inadvertent operation. The robot arm avoids such conflicting operation by exchanging the respective modality's status with the room control system. Other safety controls include limited maximum speed and rotation. At the shuttle position, the robot arm picks up the treatment table using a highly accurate attachment (tool changer) on the table top ((Fig. 4a).

*Treatment table*: Most carbon beam treatment centers have not installed a gantry beam port, which limits the irradiation beam angle that they can use. Patients should be rotated to allow treatment beam irradiation at any beam angle by rotating the treatment table. Given that the patient is fixed by immobilization with a shell, which covers the patient and is affixed with tape to the bottom of the table, a round table shape will facilitate adjustment of the shell immobilization position due to interfractional body shape changes.

We have experienced several problems with passive beam treatment, as follows. In the existing facility, for example, when hydraulic urethane resins are used to fix the head and feet ends, it is difficult for tall patients to lie on the treatment bed (= 200 cm) with feet outstretched. Further, many patients find that the narrow table width of 25–33 cm is uncomfortable. Since this treatment table is supported at both ends (head and feet ends), treatment table deflection is not particularly large, and the maximum treatment bed thickness is approximately 3.5 cm. As an additional problem, the respiratory sensing system and emergency button cables in the existing facility are on the treatment room floor, which poses a tripping danger.

The treatment table in the new facility has been changed to overcome these problems. Table length, width, and thickness have been extended to 220 cm, 40 cm (flat top), and 7 cm at the deepest, respectively ((Fig. 4d). This table thickness achieves a magnitude of table deflection under a 135 kg load of approximately 5 mm at the origin, even though the table is supported at the foot end only. The respiratory sensing system and emergency button cables can be connected directly to the table, avoiding the tripping risk of the previous cabling system ((Fig. 4c), and the table can receive attachments from both types of immobilization device (reusable and disposal) thanks to its flat top/rounded base design.

*Shuttle*: The shuttle transfers the patient from the preparation room to the treatment room. The shuttle flat top position can be changed from 50 cm to 115 cm by an auto lifting function ((Fig. 4e). Shuttle specifications are 228 cm length and 60 cm width. Handling is facilitated by an integrated power‐assist system.

### B.2 CT scanner

Several treatment centers still use a diagnostic CT scanner with a relative small gantry (typically 70cm diameter). However, these do not always allow a fully complete simulation process, as the positioning of a raised arm with immobilization conflict with CT bore installed a large‐bore 16 multislice CT (MSCT) (90 cm diameter bore size) on rails in the 2nd simulation room (Aquilion LB, Toshiba Medical Systems, Japan) ((Fig. 4c). Maximum CT acquisition distance is 1.5 m and maximum scan field‐of‐view (SFOV) is a 70 cm diameter. We use helical mode for most cases, as well as respiratory gated and 4D modes. Touch sensors are affixed on the CT front/back covers to avoid positional and operational conflict with the patient, medical staff, and other medical modalities (patient support system, irradiation ports). Although we prepared space for installation of the CT scanner in the treatment room, it was instead installed in the simulation room only. This room also contains installations of the same medical equipment as the treatment room, with the exception of the irradiation ports, for which only the irradiation covers were installed.

### B.3 X‐ray imaging system

**Figure 5:**
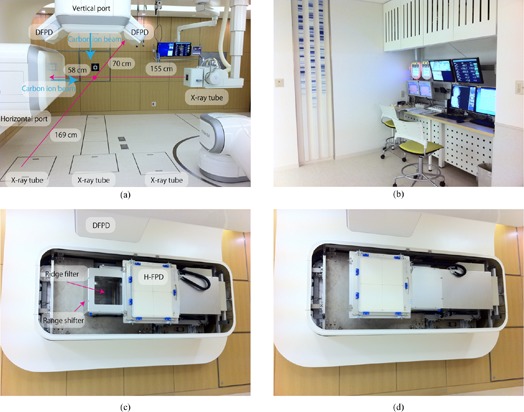
Geometry of the x‐ray imaging system (a) in the treatment room (red circle shows the room isocenter position, while blue arrows show carbon ion beam direction); (b) consoles for the X‐ray system and PTH remote access client device; (c) FPD original position; (d) FPD acquisition position.

The new system includes orthogonal (vertical and horizontal directions) X‐ray imaging systems with flat panel detectors (FPDs) for patient position verification ((Fig. 5a). All FPDs are installed within the port cover, and all X‐ray tubes except horizontal tubes are set under the floor. The horizontal X‐ray tube is set at the opposite side of the horizontal FPD and moved down when it is used ((Fig. 5a). From the patient's view, this horizontal X‐ray tube is the only movable equipment, and because it is distant from the room isocenter (ISO) and source–image receptor distances (SID) are 155 cm and 213 cm, respectively, it is unlikely to cause collision accidents. The X‐ray console is located between the room entrance and labyrinth ((Fig. 5b). For respiratory gated treatment, X‐ray images are acquired with a gating respiratory trigger from the respiratory gating system. In continuous acquisition mode, a vertical X‐ray image is alternatively acquired after the horizontal image by pressing the acquisition button once. These gated/cine images were used for patient positioning.

For the treatment room, an FPD (CXDI55C, Canon, Tokyo, Japan) with an imaging area size of 35 cm x 43 cm, pixel pitch of 160 μm, and CsI scintillator receptor is used for static image acquisition. The FPD slides to the front of the irradiation port from the original position for patient positional verification ((Figs. 5c)and (5d). For the simulation room, organ motion is checked by cine imaging using a dynamic FPD (DFPD) (DAR8000f, Shimadzu Cop. Kyoto, Japan) with an imaging area of 43 cm x 43 cm, pixel pitch of 150 μm, and aSe photoconductor, with a maximum of 30 frames per second.^(15)^ FPDs are not moved because there is no actual irradiation port.

### B.4 Respiratory gating system

Two types of respiratory gating mode are available, namely external respiratory gating and fluoroscopic internal gating modes.

External gating is generally performed using tagging points with reflective artificial markers termed “passive”.^(16)^ The process of generating software images with acquisition video images of approximately 30 Hz results in an approximately 100 ms delay in the output of gating signals.^(17)^ Because this delay might result in target irradiation errors, we routinely use the “active” sensing system, which consists of a position‐sensitive detector (PSD) sensor and infrared‐emitting light marker. Generation of a high sampling rate using a programmable logic controller results in an approximately 5 ms delay in sending the gating signals.

Use of the respiratory pattern may result in inconsistencies, particularly in patients with irregular breathing (e.g., phase shift/drift). Phase shift induces inconsistencies between external respiratory signals and internal target movements.^(18)^ Tumor position is therefore acquired continuously with or without an implanted fiducial marker using two oblique X‐ray fluoroscopic units (internal respiratory gating mode). The DFPD is installed on the right or left side of the vertical irradiation port, and the respective X‐ray tubes are installed under the floor ((Fig. 5a). ISO and SID are 169 cm and 239 cm, respectively.

### B.5 Other equipment

*Room control system*: All modalities in the treatment/simulation room have their own status (current position, turn on/off). The room control system manages these statuses to avoid conflicts and interlocking. When all modalities are at a ready for irradiation status, the room control system sends an enabling signal to the S‐IR to allow irradiation to proceed. This system, therefore, allows tracking any downtime and hardware failures in PTH system.

**Figure 6:**
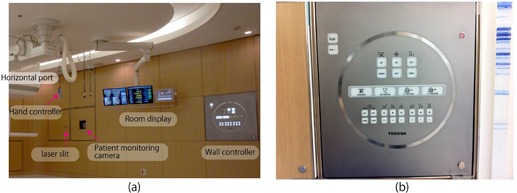
Room equipment (a) including room display, hand‐held/wall‐mounted controllers; (b) close‐up view of wall controller.

*Laser*: Several lasers are installed within the wall and provide lighting through laser slits in the wall ((Figs. 3c), 3(d) and 6(a)). Lasers cannot be installed at the inner surface of the scanning irradiating port due to presence of the range shifter. Horizontal lasers are provided at the horizontal port sides to provide horizontal reference lines at the horizontal port side.

*Hand‐held/wall‐mounted controller*: The room equipment above is controlled by hand‐held or wall‐mounted controllers. For the wall‐mounted controllers, buttons available for use under the particular configuration of the room at that time are illuminated ((Figs. 6a)and (6b). The wired hand‐held controller is operated via a touch panel TFT color display which shows a numeric keypad used to select functions, input numeric values, and display specific menus by changing display pages.

*Patient communication tool*: A patient communication tool includes a patient monitoring camera, nurse call button, and intercom. There are three patient monitoring cameras to ensure that there are no blind spots, and images are monitored by medical staff from the control room. This system is also useful to find the bottle neck for patient staying in treatment room. The medical staff can converse with the patient via the intercom.

*Data display*: Three types of data display are hung from the ceiling, namely a TMS screen, a screen for treatment table position and room equipment status information (respective room equipment movement, error messages), and a screen for treatment beam line selection.

### B.6 Software and workstation

Several custom software applications have been developed to facilitate treatment workflow. These are installed on the PTH workstations (PTHW) (Precision R5400, Dell, Roundrock, TX) assigned to respective rooms, which are housed in the server room. Medical staff control the PTHW from the treatment/simulation room using a remote access device (ELSA VIXEL D200, ELSA JAPAN, Tokyo, Japan). If the PTHW stops due to workstation error, the PTHW automatically changes to a reserve workstation and the treatment is continued in a fail‐safe manner.

*Application interface (AIF)*: Most digital files such as patient information, treatment scheduling data, and images are formatted and handled in the HL7 and DICOM formats. Not all modalities are unified under these formats, however, and a number of modality‐specific formats remained in use.

The AIF has three main functions, namely DICOM/non‐DICOM conversion, query and retrieval (Q/R) from and storage in the PACS, and data exchange between PTH modalities and others (TMS, PACS, S‐IR and TPS).

*Patient handling system application (PTHA)*: The PTHA is installed in the PTHW, which controls treatment workflow. It is extendable in response to changes in treatment workflow. Several PTHAs are installed, including a DRR application,^(19)^ patient position verification application (landmark‐based manual registration, GPU‐based 2D/3D auto‐registration),^(20)^ quality assurance application, 4D tool (image registration, and 4D dose calculation by exchange of data with the treatment planning system). These applications were custom designed for the new facility for use with any treatment workflow.

### C. Treatment workflow

Because currently available software does not always handle workflow events flexibly, we focused on designing the treatment workflow to be suitable for use with any treatment situation, such as interruption during irradiation or actual simulation and the restarting of treatment processes. The main workflow is immobilization, CT acquisition, treatment planning, simulation, quality assurance, and treatment beam irradiation. Below we describe our three most common workflows, with particular emphasis on how they differ in the sequencing of their treatment planning and simulation processes ((Fig. 2b). The TMS sends order messages to the respective systems (PTH, S‐IR, and TPS) and receives order responses in the respective workflows.

#### C.1 Preparation and immobilization

One way to improve efficiency and lower costs is to minimize occupancy time of the treatment room by minimizing the time required for patient positioning and immobilization.^(21)^ In our new facility, the patient enters the preparation room, changes into an examination gown, lies on the treatment table on the shuttle, and then undergoes any necessary preprocessing such as filling the bladder with sterilized water or emptying the rectum by enema. Medical staff then transfers the shuttle from the preparation room to the treatment/simulation room, or alternatively allow the patient to walk. By doing this, a few or several minutes, treatment room occupancy time could be minimized.

#### C.2 CT acquisition

The TMS exchanges CT examination order/response messages with the CT console by MWM (modality worklist management) and MPPS (modality performed procedure step). After the patient enters the simulation room, the treatment table is picked up by the robotic arm and transferred to the room isocenter automatically ((Figs. 3a)and (3b). The CT isocenter position is defined on the patient surface using lasers which are localized to the room isocenter. This position is recorded as the “CT isocenter setting position” in the room control system. The treatment table is then moved to the CT acquisition position and the CT moves on rails to the CT isocenter position ((Fig. 3c).

#### C.3 Treatment planning and simulation

There are three scenarios in the treatment planning simulation process.

##### C.3.1 Workflow 1

Treatment workflow 1 is a simulation (physical simulation) conducted after treatment planning (left row in (Fig. 2b). The oncologist inputs contours of the target and organs after importing CT images into the treatment planning system. The irradiation schedule is also defined here. The patient, who is lying on the treatment table, is transferred to the room isocenter to match the target isocenter position, as defined in treatment planning. The patient position verification process is performed to register the orthogonal X‐ray images to the reference DRR images. The registered FPD images are used as the reference images during the treatment course.

##### C.3.2 Workflow 2

Treatment workflow 2 is a virtual simulation conducted after treatment planning (middle row in (Fig. 2b). The process from immobilization to treatment planning is the same as that in workflow 1, with the exception that the virtual simulation is performed instead of the physical simulation. The virtual simulation is done using the planning CT data and provides orthogonal DRR images for position verification in the treatment stage.

##### C.3.3 Workflow 3

Treatment workflow 3 is performed for treatment planning after the physical simulation (right row in (Fig. 2b). While workflows 1 and 2 can be performed on a different day to the CT acquisition, workflow 3 should be done after CT acquisition while the patient remains on the treatment table. The target isocenter is generally defined using the contour data input into the treatment planning software. If the simulation process is performed on a different day from the CT acquisition, any resulting interfractional changes may prolong patient setup. In this workflow, target isocenter definition is done without the contours. Target position can be identified roughly on the planning CT images. Use of the exact same target isocenter is not necessary because if the isocenter roughly identified on the planning CT images is within a few centimeters from the target isocenter defined by using contours, simulation process can be continued by shifting the scanning beam axis.

After the isocenter is defined, the reference DRR images are calculated to verify patient position.

### C.4 Quality assurance

The quality assurance (QA) procedures for PTH and S‐IR are performed daily before treatment (not including patient‐specific QA), and take less than 15‐30 min each. QA procedures for S‐IR have been described elsewhere;^3^, ^22^, ^23^ here, we describe the daily QA procedures for PTH, as follows.

**Figure 7:**
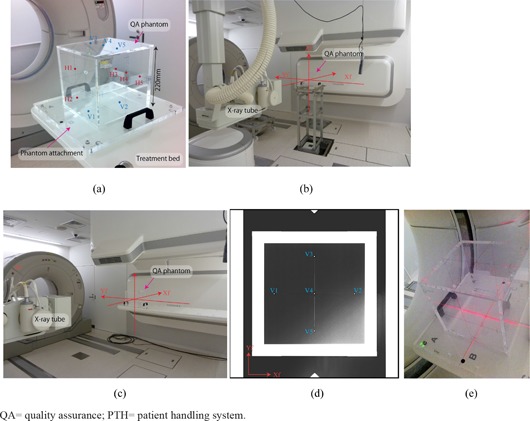
QA phantom and phantom attachment (a) (blue (V1–V5) and red points (H1–H5) are small beads on the vertical and horizontal planes); (b) QA phantom on the QA stand and (c) on the treatment table (annotations Xf, Yf and Zf show room coordinate axis defined by IEC^(14)^); (d) QA phantom X‐ray image in the vertical direction (blue points (V1–V5) are small beads positioned on the QA phantom vertical planes); (e) CT gantry laser QA.

Positional accuracy for the orthogonal X‐ray imaging system, CT scanner, robot arm treatment table, and room laser is confirmed using the QA phantom, which is an acrylic hollow box (220 mm length square, 10 mm thickness) with 10 stainless steel beads (2 mm diameter) set on the phantom plane ((Fig. 7a). At the commissioning stage, the QA phantom was placed on the QA stand and positional accuracy was adjusted using two transits, and orthogonal X‐ray images for reference images were acquired ((Fig. 7b). For the daily QA, in contrast, the QA phantom is placed on the treatment table ((Fig. 7c), and orthogonal X‐ray images are acquired ((Fig. 7d). After these X‐ray images are imported into the QA software, the QA phantom pose (position and angle) is calculated by calculating small bead positions and comparing them to those of the reference images. For laser equipment (room laser and CT laser), positional accuracy is checked by observing the laser position and QA phantom cross center lines on the phantom plates ((Fig. 7e).

QA=quality assurance; PTH=patient handling system.

### C.5 Treatment

There are three treatment rooms in the new treatment facility. The TMS sends an order message to the treatment room, where the patient is checked in by ID card at entry. This means that the patient can enter the treatment room when it is vacant to improve workflow. The patient enters by transfer on the shuttle or walking. The treatment table is transferred to the irradiation port by the robotic arm automatically. After patient position verification, the medical staff move to the control room which contains a single installation of the control system, but is nevertheless able to control all treatment rooms via a room selection function.

## III. RESULTS

The first clinical trials without respiratory gating were successfully started in the second quarter of 2011 in 11 patients with tumors of the head and neck (5 patients), prostate (3 patients), and pelvis (3 patients) who were receiving carbon ion scanning beam treatment. The patients gave informed consent to participate in the study, which was approved by the Institutional Review Board of our institute. The number of fractions was 12 or 16 for each patient, and the total treatment of fractions for all patients was 172.

As part of these trials, we also measured treatment room occupancy time in the treatment stage. Room occupancy time consists of the following steps: entry into the room (including change of clothing and immobilization), patient setup, preparation for irradiation (movement of therapists from the treatment room to the irradiation control room), irradiation, and exit from the treatment room.

**Figure 8:**
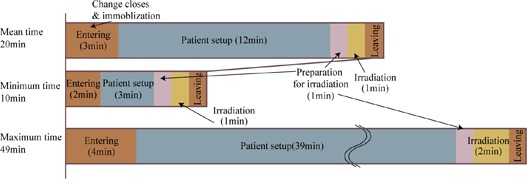
Treatment room occupation time averaged over all 11 patients.

Treatment room occupation time averaged over all patients is summarized in Fig. 8. Average occupation time was 20 min (entry, 3 min; patient setup, 12 min; preparation for irradiation, 1 min; irradiation (average target volume: 105 cc, average prescribed dose: 3.8 GyE), 1 min; and exit, 3 min). Minimum occupation time averaged over all patients was 10 min (entry, 2 min; patient setup, 3 min; preparation for irradiation, 1 min; irradiation, 1 min; and exit, 3 min).

**Figure 9:**
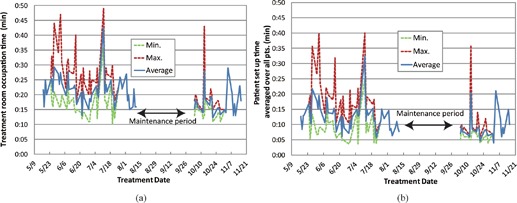
Treatment room occupation time (a) and patient setup time (b) averaged over all 11 patients as a function of treatment date.

Treatment room occupation time averaged over all patients as a function of treatment date is summarized in Fig. 9. As we scheduled a summer maintenance period of approximately 1 month, treatment was not done during that period. Average room occupation time showed minor fluctuation, but was sharply increased in a few fractions which required an extended patient setup procedure ((Fig. 9b).^(20)^ We found five cases over 30 min patient setup process during the clinical trials, this is because patients moved during patient setup process.

## IV. DISCUSSION & CONCLUSIONS

We introduced a PTH into a new treatment system. The system incorporates sophisticated treatment techniques, such as an irradiation machine, dose calculation engine, imaging/positioning machines, and others. Ongoing development is aimed at allowing responses to the treatment protocol, such as in replanning during the treatment course, hypofractionation, dose escalation, and intra‐/interfractional changes.

Most treatment centers, including ours, have long emphasized development and research for irradiation and dose calculation. Image‐guided radiotherapy has received considerable attention and is now integrated into clinical protocols.^(24)^ In particular, particle therapy has attracted considerable interest, owing to its huge construction, equipment (X‐ray imaging system, CT) maintenance, and staffing costs, and requirement for an accelerator‐equipped treatment machine. We previously developed a carbon ion accelerator which is approximately 60% smaller than that in our center, which is now installed in Gunma University, Japan.^(25)^ Several solutions to these problems have been suggested, the most important of which involve improvements in treatment workflow and direct effects on cost reduction. This is important for particle therapy, as well as photon therapy now.^(26)^ In our center, more than 700 patients per year are treated by passive irradiation therapy over 4 days per week. One of our goals is to increase capacity to meet the increasing numbers of patients with high treatment accuracy and patient comfortable.^3^, ^4^, ^27^ PTH provides several solutions to the above problems, including minimizing the number of days required for a treatment course, treatment room occupancy time, automatic patient transfer by robotic arm and shuttle, and automatic patient position verification. Here, we report treatment room occupancy time for 11 patients in our first clinical trials of carbon ion scanning beam treatment. Although this study reports our first experience with a limited number of patients in a new treatment facility, it suggests that PTH systems might be useful in particle treatment centers. We are now preparing for respiratory gating treatment to start in the near future. Further, an additional implementation of the carbon ion beam treatment system described here will be installed at Kanagawa Cancer Center, Japan. We believe that the PTH will improve treatment workflow and is suitable for use in image guided particle therapy.

## ACKNOWLEDGMENTS

We wish to thank the physics department staff and hospital staff of our institute for their support and discussion. We are also grateful to the members of Toshiba Corp, Takenaka Corp, and Nihon Sekkei Inc. for the construction of the new treatment facility.
